# Trends of emergency department visits for cannabinoid hyperemesis syndrome in Nevada: An interrupted time series analysis

**DOI:** 10.1371/journal.pone.0303205

**Published:** 2024-05-29

**Authors:** Jaeseung Soh, Yonsu Kim, Jay Shen, Mingon Kang, Stefan Chaudhry, Tae Ha Chung, Seo Hyun Kim, Yena Hwang, Daniel Lim, Adam Khattak, Leora Frimer, Ji Won Yoo

**Affiliations:** 1 Department of Internal Medicine, Kirk Kerkorian School of Medicine, University of Nevada, Las Vegas, NV, United States of America; 2 Department of Internal Medicine, Hallym University Sacred Heart Hospital, University of Hallym College of Medicine, Anyang, Korea; 3 Department of Healthcare Administration and Policy, School of Public Health, University of Nevada, Las Vegas, NV, United States of America; 4 Department of Computer Science, Howard Hughes College of Engineering, University of Nevada, Las Vegas, NV, United States of America; 5 Department of Family Medicine, Wonju Severance Hospital, Wonju, Gangwon Province, Korea; 6 Connection Sphere, Las Vegas, NV, United States of America; University of Brescia: Universita degli Studi di Brescia, ITALY

## Abstract

Cannabis-related emergency department visits have increased after legalization of cannabis for medical and recreational use. Accordingly, the incidence of emergency department visits due to cannabinoid hyperemesis syndrome in patients with chronic cannabis use has also increased. The aim of this study was to examine trends of emergency department visit due to cannabinoid hyperemesis syndrome in Nevada and evaluate factors associated with the increased risk for emergency department visit. The State Emergency Department Databases of Nevada between 2013 and 2021 were used for investigating trends of emergency department visits for cannabinoid hyperemesis syndrome. We compared patients visiting the emergency department due to cannabinoid hyperemesis syndrome with those visiting the emergency department due to other causes except cannabinoid hyperemesis and estimated the impact of cannabis commercialization for recreational use. Emergency department visits due to cannabinoid hyperemesis syndrome have continuously increased during the study period. The number of emergency department visits per 100,000 was 1.07 before commercialization for recreational use. It increased to 2.22 per 100,000 (by approximately 1.1 per 100,000) after commercialization in the third quarter of 2017. Those with cannabinoid hyperemesis syndrome were younger with fewer male patients than those without cannabinoid hyperemesis syndrome. A substantial increase in emergency department visits due to cannabinoid hyperemesis syndrome occurred in Nevada, especially after the commercialization of recreational cannabis. Further study is needed to explore factors associated with emergency department visits.

## Introduction

In 2022, drug-related emergency department (ED) visits increased, with an estimate of more than 7 million visits in the United States (U.S.) [1]. The term cannabis refers to the plant cannabis sativa and its various preparations. There are more than 60 cannabinoids that are purified from the marijuana plant, synthesized in the laboratory. Cannabis was the third most common substance drug following alcohol and opioid in 2022. Rate of cannabis in drug-related ED visits has been increasing [2, 3]. Gastrointestinal conditions, for example, nausea and vomiting were reported the most common reason for cannabis-related ED visits in nationwide ED dataset analysis [2]. Chronic cannabis use may develop increased episodes of nausea and vomiting, known as cannabinoid hyperemesis syndrome (CHS) [2]. Youths and young adults are known to be at the greatest risk of cannabis-related ED visits with CHS, a condition of composite gastrointestinal symptoms including nausea, vomiting, and abdominal pain after a chronic cannabis use [2]. Cannabis is referred to as a “gateway” drug and are concerned that marijuana legalization will advance to the hard drugs. especially, youths and young adults [3]. As of June 2023, 23 states, two territories, and the District of Columbia (DC) have legalized cannabis for adult nonmedical use and 38 states, three territories, and DC allow cannabis for medical use [4]. The State of Nevada legalized cannabis for medical use and recreational use in 2001 and 2016, respectively. Even before the commercialization of recreational marijuana, the number of individuals with cannabis-associated ED visits steadily increased by 23% annually from 2009 to 2015 in the State of Nevada [5], which has granted the authority to license and regulate cannabis consumption lounges statewide since 2022 [6]. There is little evidence of public health outcome impacts of recreational marijuana commercialization in the State of Nevada where there is a statewide provider shortage with a rapid population growth in the past decade [7]. The incidence of CHS and cannabis-related ED visits has increased significantly in states where it has been legalized [8]. The aim of this study was to examine trends of CHS ED visits after legalization of cannabis in Nevada. In addition, we evaluated factors associated with increased risk for those ED visits by comparing the characteristics of CHS patients with those of non CHS patients and estimated the impact of commercialization of recreational use of cannabis on CHS ED visits. We hypothesized that CHS ED visits increased after commercialization of cannabis for recreational use.

## Materials and methods

### Study population and dataset

The State Emergency Department Database (SEDD) of Nevada between January 2013 and December 2021 was used to identify trends of cannabis-related ED visits and CHS. The SEDD, a de-identified dataset, contains complete information on ED visits from all non-federal acute community hospitals in Nevada [9]. CHS ED visits were identified based on diagnostic codes of International Classification of Diseases, the 9th Edition, Clinical Modification (ICD-9-CM) (2013 –the 3rd Quarter of 2015), and the 10th Edition of Clinical Modification (ICD-10-CM) (the 4th Quarter of 2015 to 2021). Cannabis mental health-related disorder included those categorized under ICD-9-CM codes of 304.3 and 305.2 and ICD-10-CM codes of F12. Nausea and vomiting cases included those who were diagnosed with ICD-9-CM codes of 536.2 and 787.0 and ICD-10-CM codes of R11. We defined CHS ED visits as those who visited ED with cannabis mental health-related disorders and vomiting diagnosing codes. Since CHS is not widely recognized in real-world practice, ICD codes capturing CHS from claim data were used as described elsewhere [10–13]. Total study period was 9 years. It was divided into two periods. The first period (before commercialization of cannabis for recreational use) was from the first quarter of 2013 to the second quarter of 2017. The second period (after commercialization of cannabis for recreational use) was from the third quarter of 2017 to the fourth quarter of 2021. The following characteristics of enrolled individuals were analyzed: age, sex, race/ethnicity, use of other substances (including opioid, alcohol, smoking, and sedatives), and mood disorders histories. We identified cases with use of other substances and mood disorders with ICD-9-CM and ICD-10-CM diagnosing codes. Since the SEDD of Nevada database provides administrative data after a complete de-identification, an institutional review board approval was waived by the University of Nevada, Las Vegas.

### Statistical analyses

To identify variations of CHS ED visits after commercialization, we applied an interrupted time series (ITS) analysis by creating an aggregated time series variable by quarter [14]. This procedure generated a total of 36 time series for CHD ED visits (per 100,000) over 9 years (2013–2021). We also created an indicator coded “0” before each treatment (e.g., 2017 Q3, 2017 Q4, and 2018 Q1) and coded “1” after each treatment to examine intervention effects.

A bivariate analysis with Pearson’s chi-squared test was used to compare demographics, covariates, and outcomes of interest between CHS and non-CHS groups. Two-sided *P*-values are reported. A *P*-value less than 0.05 was statistically significant. SAS version 9.4 (SAS Institute, Cary, NC, USA) was used for all statistical analyses.

## Results

### Trends of CHS ED visits in study population

**Fig 1:**
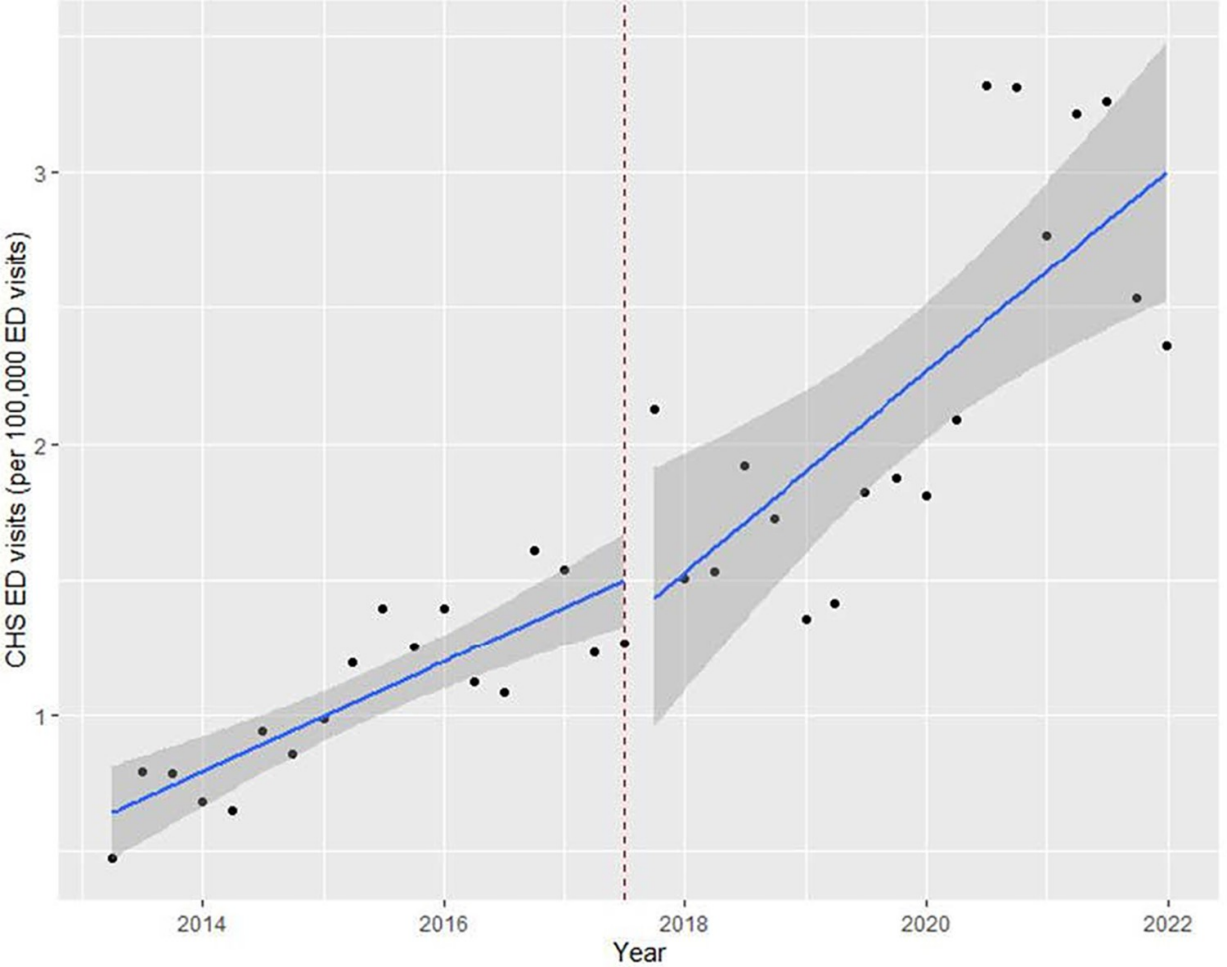
Observed and projected ED visits (per 100,000) of cannabis hyperemesis syndrome (CHS) emergency department (ED) visits in Nevada (2013–2021).

Fig 1 presents trends of observed and projected rates of CHS ED visits in Nevada, showing an increase in CHS ED visits after commercialization for recreational use in 2017. We analyzed Pre-Post data of CHS ED utilization to identify any changes after commercialization for recreational use in July 2017. The average of CHS-related ED visits was 253.1 per month before commercialization. It was increased to 263.1 after commercialization. Consistently, the number of CHS ED visits was 1.07 per 100,000 before commercialization. It increased to 2.22 per 100,000 (by approximately 1.1 per 100,000) after commercialization.

We identified a significant increase after the fourth quarter of 2017 and the first quarter of 2018 respectively. However, we found no significant results after the third quarter of 2017 when legal sales of recreational cannabis officially began. This result implies that the impact of commercialization on CHS ED visits occurred approximately after 3 months. Table 1 shows changes in CHS ED visit after commercialization for recreational use. Results of interrupted time series (ITS) analyses indicated that CHS ED visits (per 100,000) increased significantly after the fourth quarter of 2017 (Coef., 0.0006, *P* = 0.029). We also found an increased coefficient when treatment was in the first quarter of 2018 (Coef., 0.0007, *P* = 0.014). These results revealed an impact of cannabis commercialization for recreational use on CHS ED visits.

**Table 1:** Changes in CHS ED visits after commercialization.

Treatment	Parameter	Estimate	S.E.	*p*-value
2017 Q3	Intercept	-9.4358	3.6861	0.015
	Quarter	0.0005	0.0001	0.008
	Treatment	-10.5397	5.4284	0.061
	Quarter*Treatment	0.0004	0.0003	0.064
2017 Q4	Intercept	-11.4797	3.2872	0.001
	Quarter	0.0006	0.0002	0.001
	Treatment	-12.4929	5.3272	0.025
	Quarter*Treatment	0.0006	0.0003	0.029
2018 Q1	Intercept	-11.0927	3.0626	0.001
	Quarter	0.0006	0.0002	0.001
	Treatment	-14.7634	5.5905	0.013
	Quarter*Treatment	0.0007	0.0003	0.014

S.E., standard error.

### Comparison between those with CHS and those without CHS among patients visiting ED for cannabis-related disorders

We compared characteristics of CHS patients with those of non-CHS patients visiting ED during the study period (Table 2). CHS patients were younger with fewer males than non-CHS patients. The highest age group of CHS patients was 21–29 years old (35.24%) in all age categories. The CHS group had less Asian/Pacific Islander than the non-CHS group. Other races and ethnicities were not significantly different between the two groups. The use of other substances including opioid, alcohol, smoking, and sedatives and mood disorders were lower in the CHS group than in the non-CHS group.

**Table 2:** Characteristics of cannabis-related emergency department visits between those with CHS and those without CHS.

	CHS	Non-CHS	*p*-value
Age category			
Age, mean (SD)	33.47 (12.64)	35.61 (13.91)	< .0001
-20, n (%)	1,867 (12.45)	10,728 (12.04)	< .0001
21–29, n (%)	5,284 (35.24)	26,416 (29.65)	
30–44, n (%)	5,043 (33.63)	29,405 (33.01)	
45–64, n (%)	2,444 (16.30)	19,564 (21.96)	
65+	357 (2.38)	2,978 (3.34)	
Male, n (%)	7,293 (48.65)	53,716 (60.30)	< .0001
Race/ Ethnicity			< .0001
White, n (%)	8,181 (54.56)	48,204 (54.11)	
African American, n (%)	4,005 (26.71)	22,629 (25.40)	
Hispanic, n (%)	1,490 (9.94)	8,675 (9.74)	
Asian/ Pacific Islander, n (%)	226 (1.51)	1,381 (5.96)	
Native American/ Alaskan, n (%)	81 (0.54)	983 (1.10)	
Others, n (%)	780 (5.20)	5,314 (2.14)	
Opioid, n (%)	414 (2.76)	3,647 (4.09)	< .0001
Alcohol, n (%)	907 (6.05)	11,041 (12.39)	< .0001
Smoking, n (%)	5,239 (34.94)	38,190 (42.87)	< .0001
Sedatives, n (%)	75 (0.50)	1,652 (1.85)	< .0001
Mood disorders, n (%)	1,158 (7.72)	14,450 (16.22)	< .0001

CHS, cannabis hyperemesis syndrome; SD, standard deviation. *P*-value for the mean age was calculated using t test.

## Discussion

The current study investigated trends in rates of CHS ED visits from 2013 to 2021 in Nevada. We found a steady increase in rates of CHS ED visits during this period with an apparent increase after commercialization for recreational use. In addition, patients aged 21–29 years were the most common in those with CHS ED visits and females more frequently visited ED than males.

Our study correlated with previously reported trends of an increase of CHS rate among patients visiting hospitals in North America [10–13]. Recent studies have found that rates of CHS ED visits are increased after commercialization of the cannabis market [11–13]. After medical cannabinoid legalization, rates of cannabis dependency and persistent vomiting have shown an increasing trend [15]. In line with these increasing rates, ED staff in regions where legal commercialized cannabis markets exist needs greater awareness of CHS symptoms. In Nevada, ED visits due to complaints of CHS have significantly increased after the commercialization of cannabis for recreational use. CHS ED visits increased rapidly during the COVID-19 pandemic after March 2020. However, COVID-19 was not a factor causing such a rapid increase. A cross-sectional study conducted in Ontario, Canada has also found large increases in CHS ED visits during the period of the COVID-19 pandemic [13]. Because the period of COVID-19 in the current study was relatively short, it might not fully reflect the effect of COVID-19.

We evaluated factors associated with the CHS group compared with the non-CHS group. The prevalence of cannabis use disorder among those aged 12–25 appeared to increase with time since the initiation of the survey conducted by the Substance Abuse and Mental Health Services Administration [16]. In our study, 41.6% of patients with cannabis-related ED visits were in the age group of 12–29 years. This age group showed the highest prevalence of CHS. In addition, we found that CHS ED visits occurred more commonly in women than in men. Other studies about CHS have shown similar results regarding age and sex [10, 13]. Our study showed that patients using cannabis with other substances including opioid, alcohol, smoking, and sedatives were fewer in the CHS group than in the non-CHS group. Because there were few studies comparing CHS and non-CHS groups, we could not determine the exact cause for the differences between the two groups. One of explanations was that antipsychotics for treating mood disorders could control symptoms during the hyperemetic phase. For prevention and long-term management of CHS, tricyclic antidepressants or benzodiazepines were the mainstay of therapy to achieve symptom control [17]. Because CHS had various diagnostic characteristics, it might be under-diagnosed at ED. Several practical criteria such as at least weekly cannabis use for over 1-year, severe nausea and vomiting that recurs in a cyclic pattern, accompaniment of abdominal pain, resolution of symptoms after stopping cannabis, and compulsive hot baths with symptom relief have been used for identifying patients with CHS [18]. The use of synthetic cannabis might lower the incidence of CHS. Chronic stimulation of cannabinoid 1 receptors by various cannabinoids found in the natural marijuana plant plays a role in the pathogenesis of CHS. Although a case of CHS caused by synthetic cannabinoid has been reported [19], CHS might be lower in synthetic users than in natural users.

Access to recreational cannabis dispensaries plays a role in CHS and ED visits due to CHS. It has been reported that the availability of recreational cannabis dispensaries, the presence of storefront signage indicating the availability of cannabis, and signs promoting health benefits of cannabis are associated with increased cannabis use in both youths and adults [20]. Compared with adults, youths might have more probabilities of transitioning from cannabis non-users to users when they reside in recreational cannabis commercialization states [21]. As for the stigmatization concern of cannabis illegality for those who have been sent to the criminal justice system, civil liberties advocates support recreational marijuana decriminalization across states. According to a recent study, youth marijuana possession arrest rates are unchanged in marijuana decriminalization states [22]. From this evidence, we speculate that there is an urgent need of evaluating health and criminal justice outcomes in youths since the State of Nevada has a new legal establishment of cannabis consumption lounges.

The legalization of marijuana has led to an increase in cannabis use as indicated by increasing rates of positive cannabinoid immunoassay and increasing use of cannabis-related diagnosing codes at ED [3]. Cases related to synthetic cannabinoid use have been increasingly reported [23]. However, the diagnosis of synthetic cannabis is challenging because it is not currently detectable in standard urine or serum immunoassay drug screenings. Synthetic cannabinoid is much more potent than natural cannabis because it is a full agonist with a binding affinity five times higher [23]. Such stronger binding has led to numerous adverse reactions including hypertension, tachycardia, agitation, confusion, hallucination, chest pain, seizure, and syncope. High school students using synthetic cannabinoids tend to engage in risky violent, mental, and sexual behaviors [24]. Synthetic cannabinoid use and related effects are expected to increase further. Thus, healthcare providers should be aware of CHS and other potential toxicities [25]. Our study might have underestimated the prevalence of CHS ED visits because it could not consider synthetic cannabinoid use. Healthcare providers working in ED should be aware of the stakeout role of synthetic cannabis that is not usually detected by a spot urine drug screening test in ED [25].

Resource utilization in the ED and health system is expected to be affected by the study results [2, 3]. High ED use for CHS triggers congestion in the ED waiting room, public health care cost, episodic care rather than preventive and continuity of care. Hospital admission rates may also have arisen because of cannabis-related ED visits [15].

The present study has several limitations. First, we defined CHS as a combination of diagnostic codes of cannabis-related disorder and vomiting because currently there is no diagnostic code of CHS. Our study might have underestimated the burden of CHS. Second, changes of CHS incidence could be caused by a greater awareness of CHS by ED physicians and the willingness of patients to disclose cannabis use. Third, the study period in the current study was relatively short. Effects of cannabis-related ED visits were not fully evaluated especially during the COVID-19 pandemic.

In conclusion, there was a substantial increase in CHS ED visits in Nevada and a clear-cut increase after commercialization of recreational cannabis. These CHS ED visits were common in young adults and women. Further research including a large population based on administrative health databases is needed to explore trends of CHS and related factors.
